# A Greater Intrinsic, but Not External, Motivation Toward Physical Activity Is Associated With a Lower Sitting Time

**DOI:** 10.3389/fpsyg.2022.888758

**Published:** 2022-05-12

**Authors:** Samad Esmaeilzadeh, Josune Rodriquez-Negro, Arto J. Pesola

**Affiliations:** ^1^Active Life Lab, South-Eastern Finland University of Applied Sciences, Mikkeli, Finland; ^2^Department of Physical Education and Sport, Faculty of Education and Sport, University of the Basque Country (UPV/EHU), Vitoria-Gasteiz, Spain

**Keywords:** physical activity, sitting time, self-regulated questionnaire–exercise, self-determination theory, intrinsic motivation, identified regulation, introjected regulation, external regulation

## Abstract

**Background:**

Both reducing sitting and increasing physical exercise promote health but exercising more does not necessarily reduce sitting time. One reason for this non-dependency may be that different aspects of exercise motivation are differently related to sitting time. Identifying the type of exercise motivation that would also be associated with sitting time can help to reduce sitting indirectly through increased exercise, thus bringing greater benefits.

**Methods:**

The present study explored the association between quality of motivations toward physical activity with physical activity and sitting time in a total of 373 adults (age range = 23–81; women *n* = 256). The short version of international physical activity questionnaire (IPAQ) was used for measuring physical activity and sitting time. Reasons for exercising regularly were measured with the Self-Regulated Questionnaire–Exercise (SRQ-E), including four regulation subscales to assess regulation styles (i.e., intrinsic motivation, identified regulation, introjected regulation and external regulation). Confirmatory factor analysis (CFA) was used to test the four subscales of SRQ-E (latent variables) with the data obtained using AMOS v.23 (Analysis of Moment Structures). Then, structural equation model (SEM) with maximum likelihood estimates was used to test the hypothesized model.

**Results:**

The results indicated that only intrinsic motivation, but not identified, introjected or external motivation, toward physical activity predicted both physical activity and sitting time. Higher intrinsic motivation toward physical activity was associated with both higher physical activity and lower sitting time. In addition, physical activity was a mediator for lower sitting time when the source was intrinsic motivation.

**Conclusion:**

Previous studies have shown low between and within participant correlation between sitting time and physical activity, and interventions have generally failed to both increase physical activity while decreasing sitting time. The present cross-sectional results suggest that targeting increased physical activity by increased intrinsic motivation has the potential to both increase physical activity and decrease sitting time.

## Introduction

The physical activity guidelines by the World Health Organization (WHO) recommend all adults to participate in regular physical activity across the day and week (WHO, 2020). Physical activity performed at moderate-to-vigorous intensity, often in a form of physical exercise, brings particular benefits. These benefits include a lower risk for metabolic syndrome, lower blood lipids, glucose levels and blood pressure, higher bone density, a healthy body composition, as well as psychological well-being including a lower risk for depression and anxiety (Corder et al., 2009; Powell et al., 2011). However, a few adults participate in regular physical activity and exercise (Ashe et al., 2009; Corder et al., 2009; Powell et al., 2011), and this becomes worse by increasing age (Thorp et al., 2011).

The WHO also recommends all adults to sit less (WHO, 2020). More precisely, sedentary behavior is defined as waking behavior where an individual’s energy expenditure is ≤ 1.5 metabolic equivalents (METs) and lying or reclining is the predominant posture (Tremblay et al., 2017). Sedentary behavior is related with poor health outcomes such as type 2 diabetes, cancer, cardiovascular diseases and all-cause mortality even after controlling for the level of moderate-to-vigorous activity (Biswas et al., 2015; Zhou et al., 2015; Ma et al., 2017). Therefore, both physical activity and sitting time affect health in an inter-related manner. At low level of physical activity, a given reduction in sitting time is associated with greater (absolute) benefits in reduced all-cause mortality risk, whereas at a higher physical activity level the benefits of reducing sitting become smaller because of the protective role of physical activity (Ekelund et al., 2016). Therefore, reducing sitting and increasing physical activity is particularly beneficial for those who have a low physical activity level, which is the majority of population (Ekelund et al., 2016). An intriguing and direct possibility would be to replace sitting with moderate-to-vigorous physical activity. However, there are studies arguing that the nature and determinants of sedentary behavior and physical activity/exercise look different (Owen et al., 2013) and this causes physical activity interventions to be less successful in modifying sedentary time (Finni et al., 2014; Prince et al., 2014). In other words, targeting exercise behaviors seems not to directly reduce sedentary time (Finni et al., 2014; Prince et al., 2014). To develop interventions to increase physical activity and decrease sedentary time, there is a need to better understand the common and unique determinants of both.

Motivation in general, and motivation quality in particular, is a strong reason for behaviors and plays a vital role in physical activity self-regulation (Ng et al., 2012). One interesting theory showing the importance of motivation quality is organismic integration theory (OIT) which is a sub-theory of self-determination theory (SDT) (Ryan and Deci, 2002). This theory hypothesizes that the quality of motivation is more important than the quantity (Ryan and Deci, 2002). According to OIT motivation can be ranged from amotivation which is a complete lack of motivation to intrinsic motivation which is the most autonomous form of motivation. Four types of extrinsic regulation lie between amotivation and intrinsic motivation: integrated regulation (i.e., behavior integrates within one’s goals or values), identified regulation (motivation leads of a desire to attain a personally valued outcome), introjected regulation (i.e., motivation leads to avoid feeling of guilt), and external regulation (i.e., motivation arises to satisfy the demands of others) (Ryan and Deci, 2002). Introjected and external regulations represent controlling types of motivation, and identified and integrated regulations are autonomous types of motivation (Ryan and Deci, 2002). It has been shown that more autonomous types of motivation predict actual and intended physical activity behavior, as compared to more controlled types of motivation which show lesser association with physical activity (Wilson et al., 2004). Furthermore, autonomous motivation not only predicts higher levels of physical activity, but also health outcomes (Ha and Ng, 2015), and better psychological health (Ryan and Deci, 2002). In contrast, controlled motivation is not associated with physical activity behavior, yet it relates to reduced psychological health (Ng et al., 2012). Consequently, according to OIT it is possible that higher autonomous motivation rather than controlled motivation toward physical activity may persuade individuals for healthy behaviors such as being more active and less sedentary (Fenton et al., 2014; Ha and Ng, 2015). However, there is little evidence examining this in adults. Evidence of the underlying motivational determinants of physical activity and sedentary behavior will assist in designing interventions that target both behaviors.

The aim of this study is to explore whether physical activity motivation types are associated with a lower sitting time. Our research question is whether different types of motivation toward exercise mediate lower sitting time in adults.

## Materials and Methods

The data for this study was collected as part of a WELLMIE-study, which aims at exploring motivational factors related to voluntary healthy behaviors, as well as health service use in Southeastern Finnish men and women (age range = 23–81; total *n* = 373; women *n* = 256). Convenience sampling was done by sending a study introduction email to South-Eastern Finland University of Applied Sciences employee mailing lists, and to organizations and companies on Active Life Lab mailing list. Data collection was arranged with each organization separately and interested participants from a given organization were invited for data collection in groups (20 participants max per group). All participants signed an informed consent on site before starting the data collection. Data collection was done by delivering each participant an iPad where they filled the questionnaires. A researcher was available for guidance.

After removing missing data, there remained 358 participants data for analysis. Characteristics of the participants are shown in Table 1. The study protocol was accepted by the South-Eastern Finland University of Applied Sciences ethics committee in 12/2018.

**TABLE 1:** General characteristics of the study sample (total *n* = 373; women *n* = 256).

	Total	Men	Women	*p*
**Variable**	**Mean (SD)**	**Mean (SD)**	**Mean (SD)**	
Age (year)	54.9 (12.9)	54.9 (7.1)	53.7 (6.2)	0.11
Intrinsic motivation (score)	21.0 (5.2)	20.8 (5.1)	21.1 (5.2)	0.60
Identified regulation (score)	22.5 (4.3)	21.8 (4.6)	22.8 (4.1)	0.09
Introjected regulation (score)	11.6 (5.2)	11.0 (5.3)	11.8 (5.2)	0.21
External regulation (score)	5.3 (2.8)	5.8 (3.5)	5.1 (2.4)	0.08
Sitting time (min)	343.6 (169.8)	346.3 (169.2)	336.8 (171.8)	0.63
Total physical activity (METmin/wk)	3,318.7 (3,102.5)	3,109.2 (2,915.1)	3,842.5 (3,742.1)	0.21

*For total physical activity log transformed data was used in all the analysis.*

### Measures

The short version of international physical activity questionnaire (IPAQ) was used for measuring physical activity and sitting time (Craig et al., 2003). The IPAQ has been widely used for measuring physical activity and sitting time in previous studies (Quartiroli and Maeda, 2014; Maher and Conroy, 2015). This instrument is a self-administered 7-day recall questionnaire with seven items. Three different levels of physical activity including light, moderate and vigorous are assessed using 6 items of the questionnaire and one item assesses sitting time. The IPAQ has been suggested to be a reliable [intraclass correlation (ICC) = 0.81–89] and valid (criterion validity ranging; Spearman coefficient = 0.26–0.27) (Craig et al., 2003). Time in minutes for each physical activity level (walking, moderate and vigorous) was calculated per week and metabolic equivalents minutes [MET-min (Ainsworth et al., 2000)] was estimated for each level and then the tree levels were summed as the total physical activity (MET-min/wk) as follows:


Walking⁢MET-min/wk=3.3×walking⁢min×walking⁢days

Moderate⁢MET-min/wk=4.0×moderate-intensity⁢⁢activity⁢min×moderate⁢days

Vigorous⁢MET-min/wk=8.0×vigorous-intensity⁢⁢activity⁢min×vigorous-intensity⁢days

A combined total physical activity MET-min/wk was computed as the sum of Walking + Moderate + Vigorous MET-min/wk scores.

### Motivation

Self-Regulated Questionnaire–Exercise (SRQ-E) is a validated (Ryan and Connell, 1989) 16-item instrument including four regulation subscales was used to assesses regulation styles (i.e., intrinsic motivation, identified regulation, introjected regulation and external regulation) in the participants. Using this instrument, participants were asked reasons for exercising on a regular basis. Based on the present study data Cronbach’s alpha for intrinsic motivation, identified regulation, introjected regulation and external regulation were 0.86, 0.76, 0.78, and 0.85, respectively.

### Data-Analysis

Before further analysis data were checked for normality. All variables except physical activity showed normal distribution. Therefore, log transformation was conducted for physical activity data. Natural log transformation made physical activity data normal distributed. Using SPSS v.23 Pearson correlations were conducted to show the association between study variables. The following Cohen ranges were used to interpret the Pearson correlations: 0.5 < |r| strong association; 0.3 < |r| < 0.5 moderate association; and 0.1 < |r| < 0.3 small association (Cohen, 1988).

Confirmatory factor analysis (CFA) using AMOS v.23 (Analysis of Moment Structures) was used to test the four subscales of SRQ-E (latent variables) with the data obtained. Then, structural equation model (SEM) (MacCallum and Austin, 2000) with maximum likelihood estimates (Browne and Cudeck, 1992) within the hypothesized model was performed.

For evaluating the goodness of fit a two-index presentation strategy was used (Hu et al., 1995). In this method Standardized Root Mean Square Residual (SRMR) which has been suggested to be < 0.08, is coupled with at least one or more absolute indexes to show the goodness of fit. Therefore, in addition to SRMR, we used the root mean square error of approximation (RMSEA) which 0.08–1.00 showing a marginal fit while 0.06–0.08 showing acceptable; the comparative fit index (CFI) which > 0.90 showing a good fit; the Tucker Lewis index (TLI) which > 0.90 showing an acceptable fit (Meyer et al., 1982; Browne and Cudeck, 1992; Hu et al., 1995).

## Results

Participant characteristics are presented in Table 1. There were no differences between men and women in age (mean 54.9 ± 12.9 years), motivation, sitting or physical activity variables (Table 1).

Table 2 presents correlations between motivation, physical activity and sitting time. Intrinsic motivation was correlated negatively with sitting time (*r* = −0.16), and positively with physical activity (*r* = 0.26). Similarly, identified regulation was correlated negatively with sitting time (*r* = −0.12) and positively with physical activity (*r* = 0.16). There was also a small, negative, association between external regulation and physical activity (*r* = −0.10, Table 2).

**TABLE 2:** Pearson correlation between the study variables.

	2	3	4	5	6
1- Intrinsic regulation	0.8 (*p* < 0.001)	0.06 (*p* = 0.23)	−0.17 (*p* < 0.01)	−0.17 (*p* < *0.01)*	0.26 (*p* < 0.01)
2- Identified regulation		0.17 (*p* < 0.01)	−0.12 (*p* = 0.02)	−0.12 (*p* = 0.02)	0.16 (*p* < 0.01)
3- Introjected regulation			0.41 (*p* < 0.01)	0.04 (*p* = 0.50)	−0.04 (*p* = 0.45)
4- External regulation				0.03 (*p* = 0.61)	−0.10 (*p* = 0.05)
5- Sitting time					−0.24 (*p* < 0.01)
6- Physical activity					–

**FIGURE 1:**
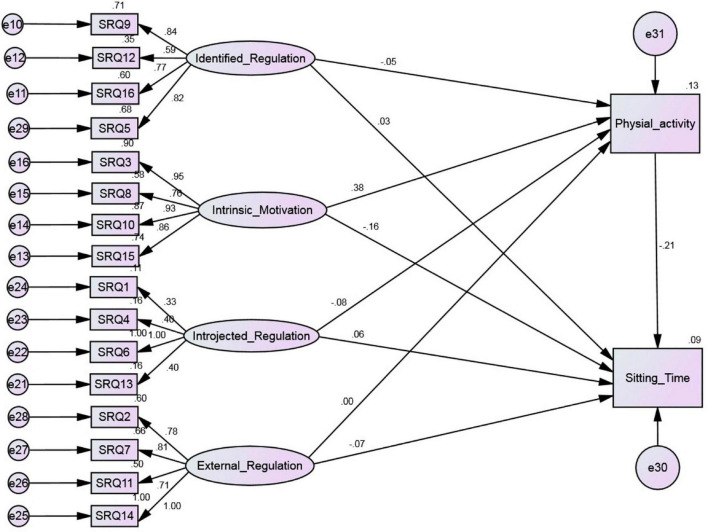
Hypothesized structural equation model predicting sitting time in adults.

**FIGURE 2:**
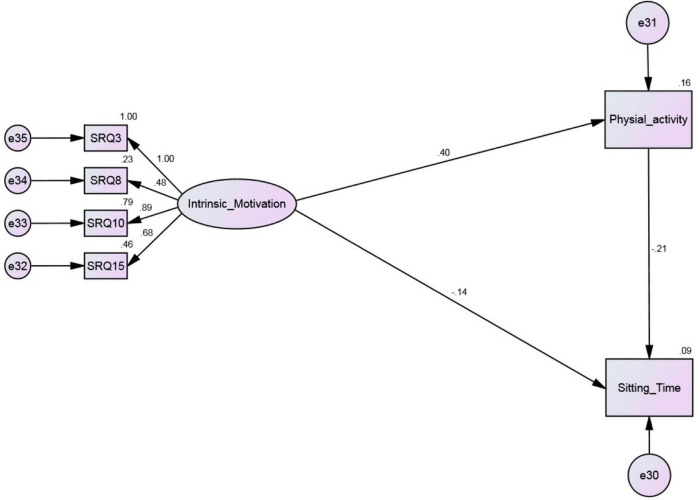
Structural equation model predicting sitting time in adults after removing non-significant associations.

Structural equation model (SEM) results are presented in Figures 1, 2. The results of CFA indicated a fit model for the four factors (*p* < 0.01; SRMR = 0.08; RMSEA = 0.08; CFI = 0.91; TLI = 0.91). For the original model (Figure 1) results revealed that the model fit was marginal (*p* < 0.01; SRMR = 0.08; RMSEA = 0.08; CFI = 0.91; TLI = 0.88). However, none of the paths that were hypothesized from identified regulation, introjected regulation and external regulation toward both physical activity and sitting time were significant and therefore were dropped from the model. The reanalyzed model (Figure 2) showed an acceptable fit (*p* = 0.06; SRMR = 0.02; RMSEA = 0.05; CFI = 0.99; TLI = 0.99). In the Figure 2 the standardized estimates are shown. Results indicated that intrinsic motivation directly and negatively predicted sitting time (β = −0.14, *p* = 0.01) but directly and positively predicted physical activity (β = 0.40, *p* < 0.01). Furthermore, the results indicated a large negative indirect effect (−0.08) of intrinsic motivation *via* physical activity to sitting time. Overall, the model suggested that physical activity mediates the association between intrinsic motivation for physical activity and sitting time (Figure 2).

## Discussion

Both increasing physical activity and reducing sitting time is beneficial for health, and therefore optimal health benefits would be obtained by replacing sitting time with physical activity (WHO, 2020). However, interventions targeting increased physical activity time generally fail to reduce sitting time, but separate intervention components are needed to reduce sitting (Finni et al., 2014; Prince et al., 2014). Physical activity and sitting time may be determined by different motivational qualities, but it has not been previously explored if different motives to exercise could mediate reduced sitting time. Therefore, the present study explored the association between quality of physical activity motivations with physical activity and sitting time in adults. The results indicated that only intrinsic motivation, but not identified, introjected or external motivation, toward physical activity predicted both physical activity and sitting time. Higher intrinsic motivation toward physical activity was associated with higher physical activity but lower sitting time. In addition, physical activity was a mediator for lower sitting time when the source was intrinsic motivation. Despite previous studies have shown low between and within participant correlation between sitting time and physical activity, the present results suggest that targeting increased physical activity by increased intrinsic motivation has the potential to both increase physical activity and decrease sitting time.

Autonomous regulations (i.e., intrinsic and identified) have been shown to be important predictors of physical activity habits. Identified regulation is a better predictor of initial adoption (short term), but intrinsic motivation is a stronger predictor of long-term exercise adherence (Ryan and Deci, 2002). Autonomous types of motivation, especially intrinsic motivation, is associated with the feelings of enjoyment, pleasure, and satisfaction. Therefore, the individual performs physical activity because he/she really likes to do it voluntarily/automatically even after the goal has been attained (Ryan and Deci, 2002; Teixeira et al., 2012). Our results are in line with these previous findings supporting the role of autonomous motivation as a positive predictor of physical activity.

By contrast, controlled types of motivation (i.e., external, and introjected regulations) which are defined as a lack of volition, are associated with goals or motives. For example, in these types of motivations the individual expects to receive something (i.e., reward) or obtains some outcome different from the activity, or to improve his/her appearance (e.g., weight management programs), or he has to do because it is prescribed, and in summary have to do/go behaviors (Ryan and Deci, 2002; Teixeira et al., 2012). These types of controlled motivations are the source of physical activity/exercise of a lot of people who are trying to be active. Previous studies reported a negative or no association between controlled types of motivation and physical activity (Teixeira et al., 2012). These types of motivations cause the behavior to be sustained only for a short time (Teixeira et al., 2012). The controlled types of motivation are associated with discomfort and anxiety in executing the behavior (Ryan and Deci, 2002). Our findings also suggest no association between controlled motivation and physical activity in adults.

The results of the present study indicated that intrinsic motivation for physical activity was negatively associated with sitting time. This suggest that adults who are intrinsically motivated for physical activity are less sedentary. According to our knowledge there is no evidence exploring the association between motivation for physical activity and sedentary behavior in adults and older people, but a few studies exist for younger people. Ha and Ng (2015) reported that autonomous motivation is associated with higher physical activity, health related quality of life, perceived physical well-being, and less sedentary behavior in students. Lonsdale et al. (2013) found that when physical education lessons are choice-based (increased autonomy) for students resulted in more physical activity but less sedentary than in the control group. Fenton et al. (2014) observed that autonomous motivation toward physical activity was negatively associated with sedentary behavior. Furthermore, they showed that controlled motivation was not associated with sedentary behavior in young adults. Quartiroli and Maeda (2014) indicated that greater autonomous motivation but not controlled motivation toward physical activity was related with lower sedentary behavior in a sample of college students. These support our findings in adults and support the importance of Self-determination theory in increasing physical activity motivation which may impact sedentary behavior.

One interesting finding of the present study was that physical activity was a mediator for lower sedentary time when the source was only intrinsic motivation. This is a novel finding and supports the idea that higher intrinsic motivation for physical activity is associated with health, and this association can be through multiple routes (also through less sitting) (Buckley et al., 2014; Rebar et al., 2016; Lachman et al., 2018).

Previous studies aiming decreasing sedentary behavior using physical activity interventions showed less success than sedentary interventions (Finni et al., 2014; Prince et al., 2014). This may happen because of using controlled motivational process which has been shown less successful in predicting physical activity behaviors (Teixeira et al., 2012). Murillo Pardo et al. (2016) found that compared to the control group an interventional program to increase perceived importance and autonomy toward physical activity had a positive impact on increasing physical activity and decreasing sedentary time in a sample of adolescents. Rollo et al. (2016) in a review paper suggested that greater support and intentions, positive attitude and greater autonomic motivation toward physical activity was a protective factor against sedentary behavior. They proposed that since there is an association between physical activity and sedentary behavior, then it is possible that physical activity related cognitions (i.e., motivation and support) could be related with sedentary time (Rollo et al., 2016). Our cross-sectional findings, testing these factors in the same model, support these findings.

It has been well established that physical activity improves quality of life (Gillison et al., 2009; Lachman et al., 2018), and interestingly, quality of life motivates the individual for physical activity (Gill et al., 2013; Lachman et al., 2018). This happens because the individuals feel that physical activity meets their needs and contribute to quality of life and as a consequence the individuals’ intrinsic motivation foster (Ryan and Deci, 2002; Lachman et al., 2018). Rebar et al. (2016) discuss that physical activity is partially regulated by non-conscious process and by pairing physical activity with a pleasant stimulus an individual may have a more favorable automatic evaluation and experience of the stimuli. They suggested that this type of non-conscious strategy may help for not only increasing physical activity but also reducing sedentary behavior (Rebar et al., 2016). Finally, Buckley et al. (2014) proposed that although external factors have an important role in facilitating either sedentary or physical activity behavior, the external factors are affected to a large degree by cognitive processes and executive functions in particular, to self-regulate an individual for future goal-directed performances to execute adaptive control anticipatorily on his/her behavior (Buckley et al., 2014). In the other words, efficient executive functions are linked with higher levels of self-regulation and this is associated with more healthful behaviors such as being more active in daily life (Buckley et al., 2014). This is supported by the observing of greater executive function which is associated with higher physical activity performance (Daly et al., 2015) but lower sedentary behavior (Buckley et al., 2014; Loprinzi and Nooe, 2016). Unfortunately, we did not measure executive function to examine these interesting associations, and we suggest for future studies to consider such measurements.

This study has limitations. It should be noted that although we included a relatively large sample size and used strict statistical methods, using questionnaires for measuring physical activity and sedentary behavior are the limitations of the study and the results need to be interpreted with caution. Future studies should use more objective measures of physical activity and sedentary time. The convenience sample may not be representative of the population and inferences can be done only related to the present sample. The study was explorative and was not powered to test a hypothesis, but these results can assist in designing a more rigor study setup, including setting a hypothesis with an appropriate sample size calculation. Optimally, decreasing sitting time through increasing physical activity intrinsic motivation should be tested in an intervention setup.

## Conclusion

The findings of the present study suggest that intrinsic physical activity motivation is associated both with a higher physical activity level, and uniquely, also with a lower sitting time. These cross-sectional preliminary findings suggest that targeting enhancing autonomous motivation, particularly intrinsic motivation toward physical activity, rather than external motivations (e.g., reward, punishment, etc.) could be used to not only promoting volitional physical activity but also to decreasing sedentary time. Further longitudinal studies are suggested to examine whether increased autonomous motivation toward physical activity can decrease sedentary behavior in adults.

## Data Availability Statement

The raw data supporting the conclusions of this article will be made available by the authors, without undue reservation.

## Ethics Statement

The studies involving human participants were reviewed and approved by South-Eastern Finland University of Applied Sciences ethics committee 12/2018. The patients/participants provided their written informed consent to participate in this study.

## Author Contributions

SE: data analysis and drafting of manuscript. JR-N: conception and design of data analysis and drafting of manuscript. AP: conception and design of experiments and data analysis, data collection, drafting of manuscript, and assisting in data analysis. All authors approved final version of the manuscript.

## Conflict of Interest

The authors declare that the research was conducted in the absence of any commercial or financial relationships that could be construed as a potential conflict of interest.

## Publisher’s Note

All claims expressed in this article are solely those of the authors and do not necessarily represent those of their affiliated organizations, or those of the publisher, the editors and the reviewers. Any product that may be evaluated in this article, or claim that may be made by its manufacturer, is not guaranteed or endorsed by the publisher.
